# Spot variation fluorescence correlation spectroscopy by data post-processing

**DOI:** 10.1038/s41598-017-05672-8

**Published:** 2017-07-17

**Authors:** S. M. J. Khadem, C. Hille, H.-G. Löhmannsröben, I. M. Sokolov

**Affiliations:** 1 Humboldt University Berlin, Institute of Physics, Berlin, D-12489 Germany; 2 Humboldt University Berlin, School of Analytical Sciences Adlershof (SALSA), Berlin, D-12489 Germany; 3 University of Potsdam, Institute of Chemistry, Karl-Liebknecht-Str. 24-25, D-14476 Potsdam-Golm, Germany

## Abstract

Spot variation fluorescence correlation spectroscopy (SV-FCS) is a variant of the FCS techniques which may give useful information about the structural organisation of the medium in which the diffusion takes place. We show that the same results can be obtained by post-processing the photon count data from ordinary FCS measurements. By using this method, one obtains the fluorescence autocorrelation functions for sizes of confocal volume, which are effectively smaller than that of the initial FCS measurement. The photon counts of the initial experiment are first transformed into smooth intensity trace using kernel smoothing method or to a piecewise-continuous intensity trace using binning and then a non-linear transformation is applied to this trace. The result of this transformation mimics the photon count rate in an experiment performed with a smaller confocal volume. The applicability of the method is established in extensive numerical simulations and directly supported in *in*-*vitro* experiments. The procedure is then applied to the diffusion of AlexaFluor647-labeled streptavidin in living cells.

## Introduction

Many concepts of fluorescence correlation spectroscopy (FCS) have been developed so far^1–4^ specially devised for particular purposes. One of them, the *spot variation fluorescence correlation spectroscopy* (SV-FCS), uses the size changes of confocal volume in order to investigate the dependence of particles’ residence time in the illuminated spot size. This provides information about the local structure of the medium in the illumination spot. Experimentally, different methods were applied to continuously vary the size of confocal volume, each having its benefits and limitations. Steinberger *et al*.^5^ measured fluorescence autocorrelation function of a planar system at different positions with respect to the confocal volume in *z*-direction with step size of typically 100–200 nm (*z*-scan). Changing the position of the planar system with respect to the beam’s waist led to change in the illuminated spot size. Assuming the quadratic dependence of residence time and number of particles inside the confocal volume on the diameter of the spot, one could measure the diffusion coefficient, the beam’s waist, and the particles’ concentration. Although this method needs no additional calibration steps, it is limited to 2D-systems. The main error sources in this method are inaccurate positioning of the sample, thermal instability during subsequent measurements, and deviation from the assumed beam’s form^6^.

Eggeling *et al*.^7^ coupled FCS with stimulated emission depletion (STED) microscopy in order to lift diffraction limitations for confocal volumes (STED-FCS). An interesting feature of STED is its ability to tune the size of confocal volume by increasing the power of the depletion laser. Performing the calibration of the intensity of laser with respect to the resulting size of the confocal volume, one can in principle reach the desired size of confocal volume in three dimensions. However, this calibration may not be accurate and therefore introduces errors in results of analysis. Moreover, higher intensities of the depletion laser for smaller spots may lead to deviations from the Gaussian intensity profile in the confocal volume which is assumed in further data processing procedures^8^. This technique is in a wider use but demands for a sophisticated setup and high-power lasers, which could be considered as disadvantage.

Another method, the spot-variation FCS (SV-FCS), was developed independently by Masuda *et al*.^9^ and Wawrezinieck *et al*.^10^ and is based on the under-filling of the back aperture of the objective by the laser beam. Wawrezinieck *et al*. introduced a diaphragm between laser beam expander and the dichroic mirror in order to select the extension of beam on the back aperture of the microscope objective. Using the direct dependence of the point spread function on the diaphragm aperture, they could tune the size of confocal volume. Masuda *et al*. also motorized the variable beam expander to adjust the size of incoming laser beam before objective. With these techniques one can continuously adjust the spot size in the range of 0.2 *μ*m up to 0.5 *μ*m.

In all methods mentioned above one has to perform many repetitions of the experiments. In each repetition the confocal volume has to be adjusted mechanically or electronically, which may lead to errors and artifacts in measured FCS curves. Thus, the size of the confocal volume is controlled only indirectly by considering its theoretically assumed dependence on the parameters changed, which can be altered due to minor deviations in the setup. Repeating the experiment many times can be a source of artifacts due to thermal instability of the system’s environment or to possible changes in the sample itself. This can be a serious issue when considering experiments in living cells which have to be exposed to laser illumination for a long time.

In what follows we introduce a new method providing the possibility of mimicking the variation of the spot’s size with any desired step size on the level of data post-processing, using the data from a single run of the standard FCS measurement with diffraction-limited confocal volume. Using the linear relationship between the intensity of the excitation laser beam and the resulting florescence intensity, the assumption which is already made in the standard FCS procedure, we apply a non-linear transformation to the estimates of the fluorescence intensities made from the data, and by this mimic the exclusion of the outer part of confocal volume from the FCS calculation. Since only a single FCS measurement is required, all challenges arising from repeating the measurements are excluded. There is no need for extra calibration and no more assumptions than have been made for the FCS measurement. Since this method uses a common FCS setup, there is also no need for extra equipment. We discuss the method in a three-dimensional setup, although it can also be applied in planar, two-dimensional systems.

## Results and Discussion

### Theory of post-processing

In a standard FCS measurement, the size of the confocal volume is defined by the properties of the laser beam and of the optical setup. The excited fluorophores within the confocal volume emit light (at a wavelength different from that of the exciting laser) which is collected by detector. The intensity of emitted light depends on the laser intensity profile *W*(**r**) and on the local fluorophore concentration *c*(**r**, *t*),

1 $$I(t)=\varepsilon \int d{\bf{r}}W({\bf{r}})c({\bf{r}},t)$$

where $\varepsilon$ denotes the total quantum efficiency of fluorescence. Autocorrelation of recorded intensities leads to the FCS curves, *G*(*τ*), which can be normalized according to

2 $$G(\tau )=\frac{\langle I(t+\tau )I(t)\rangle }{{\langle I(t)\rangle }^{2}}-1.$$

Note that due to the normalization all the pre-factors in the intensity cancel out.

The standard FCS method can be considered as a single particle technique: the measurements are preformed at very low fluorophore concentrations so that one can safely assume that there is no more than one fluorophore particle in the confocal volume at each instant of time. Therefore the instantaneous concentration *c*(**r**, *t*) in Eq. 1 is essentially *δ*(**r** − **r**(*t*)) where **r**(*t*) is the instantaneous particle’s position. The instantaneous fluorescence intensity at time *t* is thus

3 $$I(t)=\varepsilon W({\bf{r}}(t)).$$

The laser beam excitation profile *W*(**r**) is commonly approximated by a Gaussian form

$$W({\bf{r}})={I}_{0}\,\exp \,[-2\frac{{x}^{2}+{y}^{2}}{{r}_{0}^{2}}-2\frac{{z}^{2}}{{s}^{2}{r}_{0}^{2}}]$$

with *I*_0_ being the characteristic intensity, *r*_0_ being the diameter of the beam’s waist in the direction perpendicular to the light propagation direction, and *sr*_0_ being the corresponding dimension in the light propagation direction, so that

4 $$I({\bf{r}}(t))=\varepsilon {I}_{0}\,\exp \,[-2\frac{{x}^{2}(t)+{y}^{2}(t)}{{r}_{0}^{2}}-2\frac{{z}^{2}(t)}{{s}^{2}{r}_{0}^{2}}].$$

Here the origin of coordinates is put to the center of the confocal volume. In a typical FCS experiment the parameters of the beam are fixed and known in beforehand from calibration.

Calculation the intensity autocorrelation function, Eq. 2, for the intensity $I(t)=\varepsilon W({\bf{r}}(t))$ leads, under the assumption the Gaussian form of tracers’ displacements probability density function, to the standard formula for the FCS autocorrelation function for normal and for Gaussian anomalous diffusion:

5 $$G(t,\alpha ,{D}_{\alpha })={(\frac{4{D}_{\alpha }}{{r}_{0}^{2}}{t}^{\alpha }+1)}^{-1}\,{(\frac{4{D}_{\alpha }}{{s}^{2}{r}_{0}^{2}}{t}^{\alpha }+1)}^{-\frac{1}{2}}$$

where *α* is the exponent of anomalous diffusion; the case of normal diffusion corresponds to *α* = 1^11^. Note that the logarithm of *I*(*r*(*t*)) as given by Eq. 4 is immediately related to the position of particle with respect to the center of confocal volume. Therefore, the main idea of our approach to mimic the change in the size of confocal volume is to exploit the correspondence between the distance of particle from the center of the confocal volume and recorded intensity *I*(*t*). Introducing the modified intensity

6 $$I^{\prime} (t)=I(t)\,\exp \,(\frac{\mathrm{ln}\,I(t)}{{r}_{c}}),$$

with *r*_*c*_ being the free parameter of the transformation, one gets

7 $$I^{\prime} (t)=\varepsilon {I}_{0}\,\exp \,[\mathrm{ln}(\varepsilon {I}_{0})/{r}_{c}]\times \exp \,[-2\tfrac{{x}^{2}(t)+{y}^{2}(t)}{{r}_{0}^{2}}\,(\tfrac{{r}_{c}+1}{{r}_{c}})-2\tfrac{{z}^{2}(t)}{{s}^{2}{r}_{0}^{2}}\,(\tfrac{{r}_{c}+1}{{r}_{c}})].$$

The intensity *I*′(*t*) mimics a recorded intensity from a smaller confocal volume with the waist size

8 $${r}_{0}^{^{\prime} }=\sqrt{\frac{{r}_{c}}{{r}_{c}+1}}{r}_{0}$$

and with the same elongation parameter *s*. In Eq. 8, when *r*_*c*_ is taken to be large, ${r}_{0}^{^{\prime} }$ tends to *r*_0_ and when *r*_*c*_ is small, ${r}_{0}^{^{\prime} }$ is reduced by the factor $\sqrt{{r}_{c}}$. When calculating the autocorrelation according to Eq. 2 the prefactor in Eq. 7 cancels out due to normalization. In essence, all information required is the intensity from a single measurement run, there is no need to repeat the measurement, and therefore no errors due to thermal instabilities, drift, or repeated re-calibration. Taking the intensities from a single measurement and treating them with Eq. 6 for different *r*_*c*_ one can calculate the autocorrelation functions for any size of confocal volume starting from *r*_0_ down to zero (corresponding to the size of tracer) with any desired step size. The realistic situation is however somewhat more complicated, since our Eq. 6 is applicable to the intensity as a continuous function of time (the photon counting *rate*) which will be called intensity trace in what follows, and not to single photon counts, which are the typical outcome of the measurement run.

In typical FCS measurements the FCS curves are calculated from the photon arrival times^12^. Thus, the intensity profile is a binary file containing zero or one photon count per detection bin. For our approach, however, this shot noise is very disturbing. In this case, the proposed method would not work and applying the non-linear transformation would lead to the same FCS curve as the initial one. Here, we discuss two ways to circumvent the problem. The immediate idea is to use larger binning time and work with this piecewise-continuous intensity profile. Our investigations indicate that having the intensity profile varying from zero to roughly 20 photon counts per bin is sufficient to perform our analysis. This method works perfectly in the situations when the diffusion process is slow, and the diffusion time through the spot is much longer than the binning time necessary to achieve the required accuracy. The other way to overcome the problem of shot noise is to transform the initial binary intensity profile into the continuous fluorescence intensity trace (photon counting rate) which considerably varies in time according to the position of the tracer. We tried several approaches based on kernel smoothing and on the statistics of the inter-event times, and found that smoothing with the double-sided exponential kernel gives the best results among all tested variants. Thus, our estimate for the intensity is

9 $$I(t)=1/2\sigma \sum _{i=0}^{N}\,\exp \,(-|t-{t}_{i}|/\sigma )$$

where *t*_*i*_ are the photon arrival times, *N* is the total number of records during the measurement time *T*, and *σ* is the effective smoothing time which also defines the temporal resolution. The optimal value of *σ* depends on the total number of photons collected from the particle diffusing inside the confocal volume, and has to be taken such that the autocorrelation function *G*′(*t*) calculated from the reconstructed intensity trace *I*′(*t*) for ${r}_{c}\gg 1$ (i.e. when $I^{\prime} (t)\to I(t)$) shows the minimal deviation from original FCS curve *G*(*t*).

In what follows, we first discuss the method at the simulation level, emphasising the kernel smoothing approach. The suitability of the proposed method is then investigated experimentally for two cases of normal and anomalous diffusion of AlexaFluor647-labeled streptavidin in pure phosphate-buffered saline (PBS) and in PBS crowded with 30% PEG1500 which are known to be homogeneous. In this test experiments, we used both approaches, namely binning and kernel smoothing. The method was then applied to study the diffusion process in a binary mixture of dimethyl sulfoxide (DMSO) and glycerol known to form nano-domains. Finally, the method was successfully applied to living cells by studying the intracellular diffusion of AlexaFluor647-labeled streptavidin in cells of salivary gland tissue of the American cockroach *Periplaneta americana*.

### Simulation and choice of parameters

To support our theoretical finding by means of simulation, we have performed simulations for the cases of normal and anomalous diffusion. The trajectories of each tracer were recorded up to 10^8^ time steps. The time step of simulation corresponds to 1 *μ*s in a real experiment. The value of the Hurst parameter for anomalous diffusion was *H* = 0.37, corresponding to sub-diffusion with the exponent of anomalous diffusion *α* = 0.75. Diffusion coefficients were set to be 11.8 *μ*m^2^/*s* for normal and 80.1 *μ*m^2^/s^0.75^ for anomalous diffusion. The size of the simulation box was taken to be *L* = 4 *μ*m in each direction, with periodic boundary conditions. The parameters of the confocal volume in Eq. 4 were taken to be *r*_0_ = 0.3 *μ*m, and *s* = 4, the center of the confocal volume positioned in the center of the simulation box. The characteristic intensity *I*_0_ was taken to be unity. The total number of particles was chosen such that their mean number within the confocal volume did not exceed 0.5.

**Figure 1:**
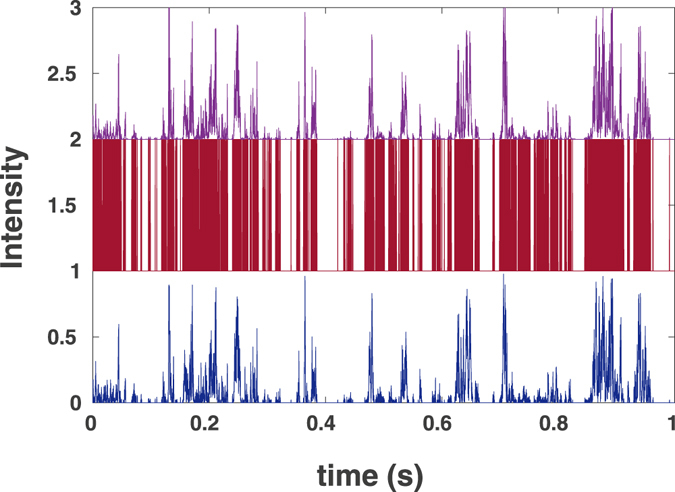
Simulation results for fluorescence intensities for the case of normal diffusion: (upper, purple) the original intensity trace *I*_0_ shifted up by 2 units; (middle, red) photon counts (*I*_*p*_) shifted up by 1 unit, and (lower, blue) restored intensity trace *I*(*t*) with *σ* = 23 *μ*s.

**Figure 2:**
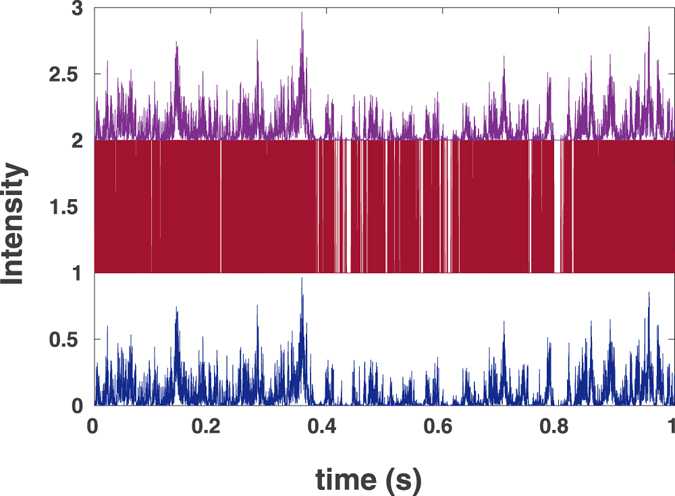
Simulation results for fluorescence intensities for the case of anomalous diffusion: (upper, purple) the original intensity *I*_0_ shifted up by 2 units; (middle, red) photon counts (*I*_*p*_) shifted up by 1 unit, and (lower, blue) restored intensity trace *I*(*t*) with *σ* = 12 *μ*s.

The simulated particles’ trajectories and the known form of the confocal volume allows for generating the intensity traces *I*_0_(*t*), see Figs 1 and 2 for normal and for anomalous diffusion, respectively. These intensities are used to simulate the photon counts *I*_*p*_ (being 0 or 1 at each time step Δ*t* with probabilities $1-\varepsilon {I}_{0}(t){\rm{\Delta }}t$ and $\varepsilon {I}_{0}(t){\rm{\Delta }}t$, respectively, with $\varepsilon =1$.

In the next step we transform the binary data to the continuous intensity trace, *I*(*t*) (Figs 1 and 2 lower (blue)) by using the Eq. 9 with *σ* = 23 *μ*s for the normal and *σ* = 12 *μ*s for anomalous diffusion. These values of parameters were chosen to minimize the deviation between the original and the restored curve.

Note that in the real experiment we do not have access to the original intensity trace *I*_0_(*t*), but only to the photon counts, here, finding the optimal value of *σ* can be done by comparing the FCS curves *G*(*t*) and *G*′(*t*) as calculated from the photon counts and from the smoothed intensity trace in the relevant time domains. Here, we compare both the intensity traces and corresponding autocorrelation functions. The aim is to show that if the autocorrelation functions from binary data and from the reconstructed intensity coincide, the initial and the reconstructed intensity traces are indeed the same to a high accuracy.

**Figure 3:**
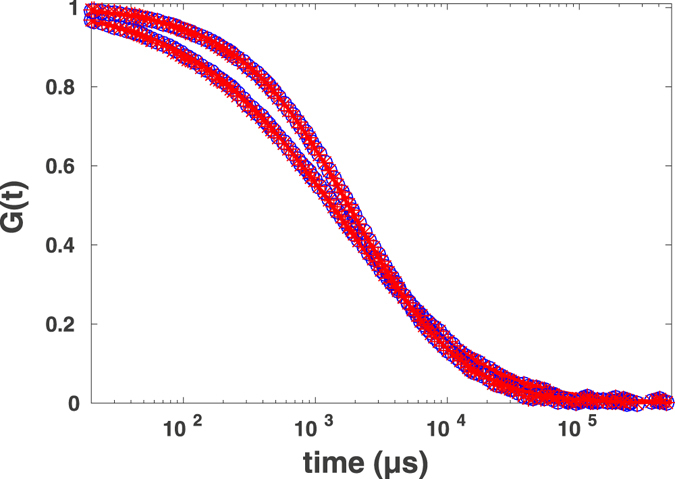
Simulation results for FCS curves calculated from photon counts (blue circles) and from restored intensity traces (red asterisks) fitted to Eq. 5 (lines) for normal (upper line) and anomalous (lower line) diffusion. Note the almost complete coincidence of data.

Figure 3 shows the autocorrelation functions calculated from the photon counts and from the restored intensities for normal and for anomalous diffusion. Figure 3 indicates that both in the normal and in the anomalous diffusion cases the autocorrelation functions of reconstructed intensities reproduce the autocorrelation functions of original data at longer time lags. We note that for the lag times of the order and below *σ* values the deviations may increase considerably. Given typical times between photon counts in real experiments this limitation is not an important issue, however, care must be taken when fitting the corresponding curves: time lags below *σ* must be excluded from the fit.

**Figure 4:**
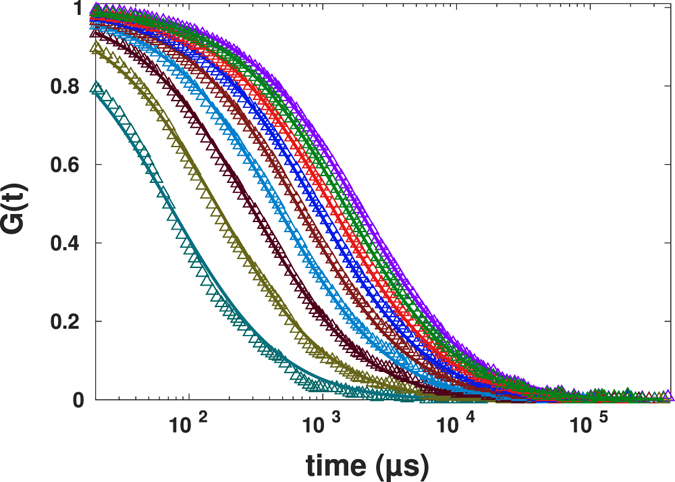
Simulation results for mimicked spot variation FCS for the values of the effective beam waist ranging from *r*_0_ to 0.2*r*_0_ (from right to left) for normal diffusion (symbols), together with theoretical FCS curves for the same beam waists according to Eq. 5.

**Figure 5:**
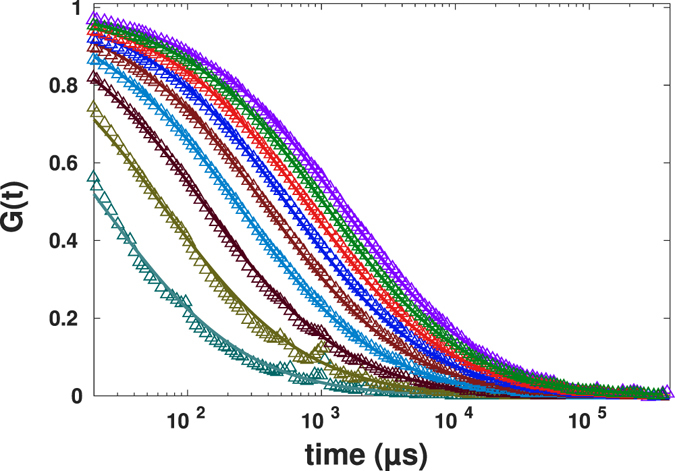
Simulation results for mimicked spot variation FCS for the values of the effective beam waist ranging from *r*_0_ to 0.2*r*_0_ (from right to left) for anomalous diffusion (symbols), together with theoretical FCS curves for the same beam waists according to Eq. 5.

In order to apply the proposed method to mimic the change of the size of confocal volume, we used Eq. 6 with changing parameter *r*_*c*_ such that the size of the resulting confocal volume decreases from initial size *r*_0_ to 0.2*r*_0_ with steps of 0.1*r*_0_, both for normal diffusion, Fig. 4, and for anomalous diffusion, Fig. 5.

Plotted are the initial FCS data for the beam waist *r*_0_, the results for the beam waists *r* < *r*_0_ as obtained by the procedure discussed, and the theoretical curves, according to Eq. 5, as calculated for the corresponding beam waists and for the known values of the coefficient of the normal or anomalous diffusion. Note that the curves in Figs 4 and 5 *are not fits*, but the theoretical curves showing the behavior given by Eq. 5 for the values of parameters used in simulations. Additionally, the simulated data were fitted to Eq. 5 to obtain the exponents of anomalous diffusion and diffusion coefficients. Results of fittings in all data for diffusion coefficients and exponents of anomalous diffusion being *D* = 12.2 ± 1.2 *μ*m^2^/s and *α* = 0.99 ± 0.01 for normal diffusion and *D* = 78.5 ± 4.8 *μ*m^2^/s^0.75^ and *α* = 0.75 ± 0.03 for anomalous diffusion, i.e. practically do not deviate from the values which were used for the trajectories’ simulation.

These findings give the direct support of the method. In what follows, we will apply the approach to the experimental data. This will give additional support for the applicability of the method and show its potential usefulness for investigations of biological systems.

### *In vitro* experiments: normal and anomalous diffusion in homogeneous media

To investigate our new method in situations of normal and anomalous diffusion, we performed FCS measurements for the cases of diffusion of 0.5 nM AlexaFluor647-labeled streptavidin in PBS buffer solution and in PBS buffer solution crowded by 30% PEG1500. We chose a low concentration of fluorescence tracer to ensure that at any time there would be on the average no more than one tracer molecule in the confocal volume. Generally, performing a single molecule FCS measurement, one should observe strong fluctuations of count rates arising from entering and exiting the tracers. In our experiments, the photon count rate in background was roughly 1,000 cps and changes to maximum roughly 10,000 cps depending on the trajectories intersecting different parts of confocal volume. To filter out the unwanted background photons coming from reflection, time gating was used.

**Figure 6:**
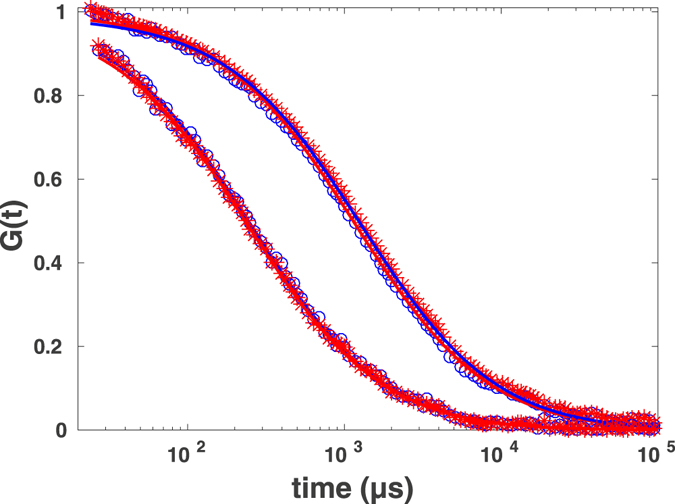
Experimental results for FCS calculated from photon counts (blue circles) and from the restored intensity trace according to Eq. 9 (red asterisks) for streptavidin diffusion in pure PBS (lower line/symbols) and in PBS crowded with 30% PEG1500 (upper line/symbols) fitted to the standard formula (solid lines). Each curve is representative for *N* = 6 independent experiments.

Autocorrelation functions for the corresponding cases calculated from recorded photon counts and from the restored intensity profiles according to Eq. 9 with *σ* = 3 *μ*s are shown in Fig. 6.

The fit for the case of crowded buffer with 30% PEG1500 led to the exponent of anomalous diffusion and diffusion time being 0.89 ± 0.03 and 1,084.3 ± 38.6 *μs*. The case of diffusion in pure buffer solution were fitted to the normal diffusion and resulted in diffusion time being 243 ± 20 *μ*s. The differences between fits to original FCS data and reconstructed ones using kernel smoothing method for both normal and anomalous diffusion were very small and here we reported the fit for smoothed curves. The standard deviations were calculated from *N* = 6 repeated experiments for both. To avoid considering the photophysics of AlexaFluor647 dyes, only lag times longer than 20 *μ*s were taken into account in the analysis of the FCS curves. However, since the diffusion processes are slow, this does not impact our analysis significantly. Note that this lag time is of the order of the time limitation of our method as defined by the value *σ* used. Similar to our simulations in the previous section, the FCS autocorrelation functions as calculated from the photon counts and from the restored intensity traces coincide (compare blue and red solid lines in Fig. 6).

**Figure 7:**
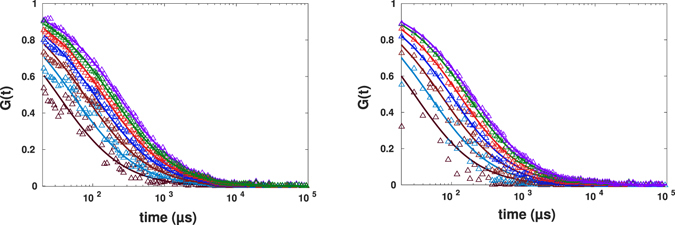
Experimental results for spot variation FCS (with the effective beam waist varying from *r*_0_ to 0.4*r*_0_ from right to left) for diffusion of AlexaFluor647-labeled streptavidin in buffer fitted to the standard formula. The left panel corresponds to intensity traces generated using kernel smoothing method, the right one to the ones obtained by binning.

**Figure 8:**
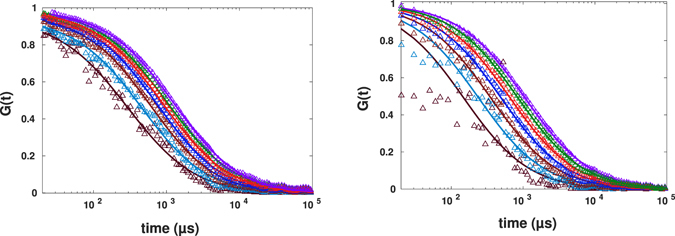
Experimental results for spot variation FCS (with *r*_0_ − 0.4*r*_0_ from right to left) for diffusion of AlexaFluor647-labeled streptavidin in buffer crowded by 30% PEG1500 fitted to the standard formula. The intensity time traces were generated using kernel smoothing method for the left panel and binning for the right panel.

To investigate the binning method, original binary photon counts were also binned with a bin width of 10 *μ*s, which results in an intensity profile varying from zero to maximum of 20 and 31 counts for normal and anomalous diffusion, respectively. Having both intensity traces at hand, we could apply the nonlinear transformation according to Eq. 6 to these restored intensity traces and mimic the spot variation FCS. Results of applying Eq. 6 to these intensity traces for the cases of pure buffer and crowded one are shown in Figs 7 and 8.

Both approaches, kernel smoothing (left) and binning (right) in both experiments revealed similar results, and the autocorrelation curves decayed faster as the spot size becomes smaller. The FCS curves generated from binned intensity traces have less data points in the first decade (10 *μs* − 100 *μs*) which could lead to more uncertainties in fitting process. On the other hand, binning has the advantage of fast analysis and does not require a thorough choice of smooting parameter required by the kernel smoothing method. The key advantage of kernel smoothing method is its applicability in case of low photon counts (arising from e.g. low laser power or low fluorescence quantum yield of dye). In such cases, to obtain a sufficient varying intensity trace from binning method, one would have to make the bin larger, leading to reduced time resolution.

The noisier data for smaller sizes of spots are due to fairly short measurement duration. For *in*-*vitro* measurements, one could simply increase the measurement time and obtain quite smooth curves also for smaller spots. We did not do so, and kept the data aquisition time to be 100 *s* in all experiments reported to comply with limitations of *in*-*vivo* experiments, where longer illumination times harm the cell.

In order to obtain a quantitative analysis of the results, we studied the phenomenological relation, ${\tau }_{d}={t}_{0}+{r}_{0}^{2}/4{D}_{eff}$, termed FCS diffusion law, suggested and investigated in simulation by Wawrezinieck *et al*.^10^. The offset time *t*_0_ vanishes in the case of free diffusion in a homogeneous medium. The analysis of Wawrezinieck *et al*.^10^ states that the existence of barriers in the confocal volume would lead to non-vanishing negative *t*_0_. On the other hand, if there is a nano-domain in the confocal volume, in which the tracer molecule is trapped for longer times, one would expect positive *t*_0_. Thus, the zero intercept, namely *t*_0_ = 0, in this analysis is the proof for homogeneity of the system. Therefore, performing this analysis one could in principle obtain valuable information about the structural conformation of the system at the nano scale.

**Figure 9:**
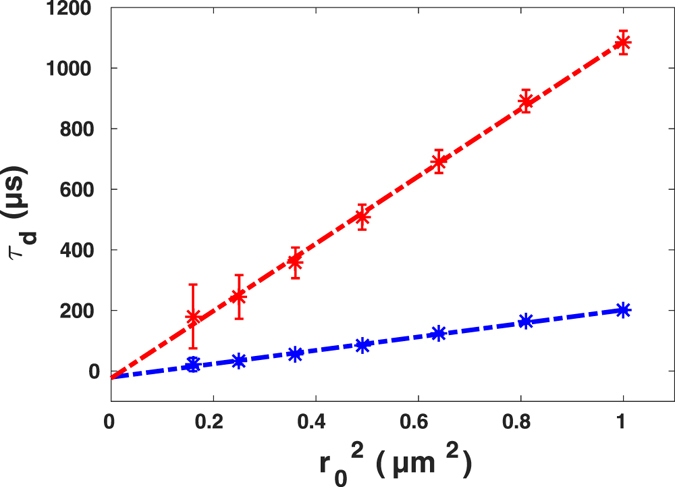
Diffusion time of AlexaFluor647-labeled streptavidin in buffer (blue) and in buffer crowded with 30% PEG1500 (red) versus spot size, together with corresponding linear fits being ${\tau }_{d}=222{r}_{0}^{2}-11$ with *Pearson r* ^2^ = 0.995 and ${\tau }_{d}=1,111{r}_{0}^{2}-23$ with *Pearson r* ^2^ = 0.997. Data are presented as means ± standard deviation from *N* = 6 independent experiments.

Here, we did the same analysis using our post-processing approach. Curves for different known sizes of confocal volume were fitted to Eq. 5 with the exponent of anomalous diffusion acquired from the initial fit to the autocorrelation function of the data (i.e. for the original size of confocal volume equal to 1.0*r*_0_). Figure 9 shows the result of the diffusion law analysis for the FCS curves shown in Figs 7 and 8.

Linear fits resulted in the intercept *t*_0_ being −11 ± 13 *μs* and −23 ± 54 *μs* for pure buffer and PEG-crowded solutions. Larger error bars in results for diffusion in buffer solution crowded by 30% PEG solution were due to including the exponent of anomalous diffusion as a free fit parameter. The parameter *α* has a standard deviation of 0.03; this small variation in exponent of anomalous diffusion has, however, a larger impact in the diffusion time calculation. In summary, these results show that in fact no barrier or domain is present in these homogeneous solutions and this is a strong witness in favor of the applicability of our approach to experimental data to prove the homogeneity of a structure concerning nano-domains.

### *In vitro* experiments: Heterogeneity in binary mixtures of dimethyl sulfoxide and glycerol

Dimethyl sulfoxide (DMSO) solution is a homogeneous system with uni-modal diffusion time distribution. However, binary mixture of DMSO and glycerol has been reported to be extremely heterogeneous. Broad range of nano- and micro-domains are created by glycerol with considerably higher viscosity. Therefore, diffusion in such system takes place with a broad range of diffusion time distribution in FCS measurements^13^.

**Figure 10:**
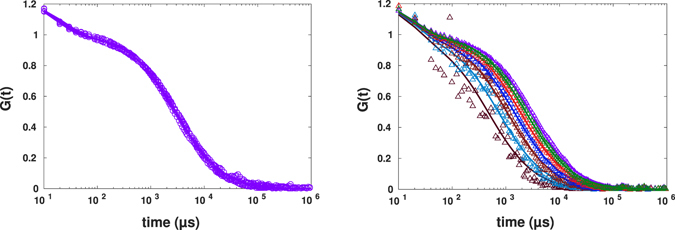
Experimental results of FCS measurements (left) for diffusion of AlexaFluor647-labeled streptavidin in pure DMSO fitted to the standard formula including triplet dynamics. Spot variation FCS with post-processing (*r*_0_ − 0.4*r*_0_ from right to left). The intensity time traces were generated using binning method (right).

**Figure 11:**
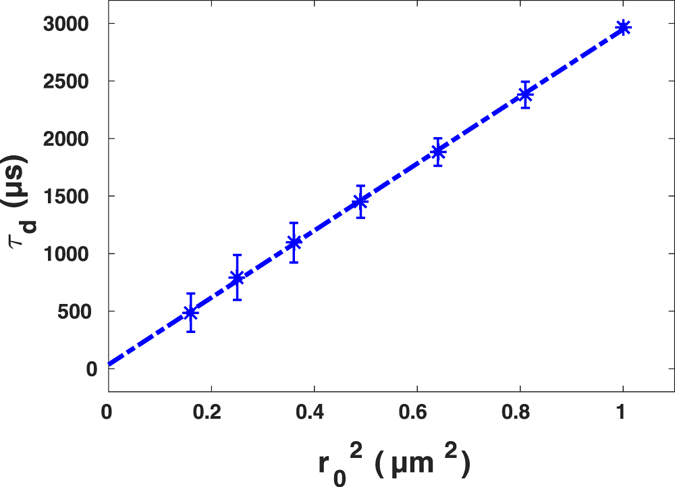
Diffusion time of AlexaFluor647-labeled streptavidin in pure DMSO versus size, together with linear fit of ${\tau }_{d}\,=\,2,912{r}_{0}^{2}\,+\,35$ with *Pearson r* ^2^ = 0.999. Data are presented as means ± standard deviation from *N* = 4 independent experiments.

Such a system provides an ideal test for the ability of our method to unravel local inhomogeneities. Compatible with other experiments in this work, we chose AlexaFluor647-labeled streptavidin tracers and performed the FCS measurements with tracer concentration being 0.5 *nM*. We started at first with the homogeneous system, being pure DMSO, and performed FCS measurements for four independent experiments, shown in Fig. 10 (left). In all experiments we obtained similiar FCS curves as expected for a homogeneous system, resulting in a normal diffusion with uni-modal diffusion time distribution with a mean value being 2,969 ± 25 *μs*. For analysis of FCS curves, we have included the triplet dynamics into Eq. 5, as previously reported for AlexaFluor647^14, 15^. In modelling the spot variation in these set of experiments, we used the binning method since the tracer diffusion is quite slow. As it is shown in Fig. 10 (right), the FCS curves corresponding to the smaller spot sizes decay faster. The diffusion law analysis shown in Fig. 11 indicates the intercept of linear fit to the diffusion time being 35 ± 133 *μs* confirming our previous knowledge on the homogeneity of the system.

**Figure 12:**
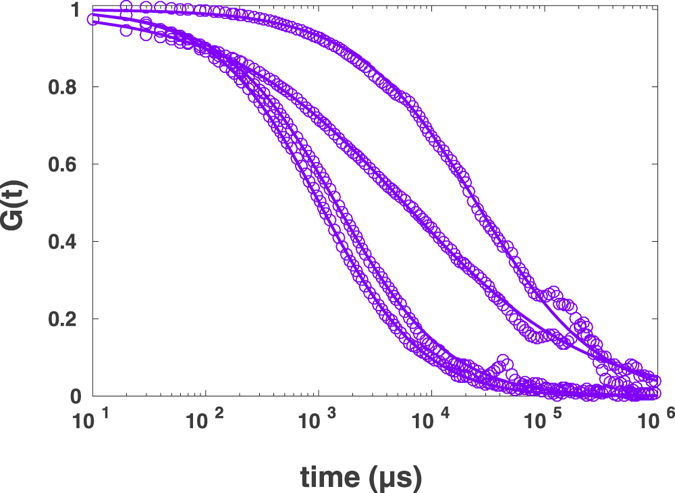
Experimental results of FCS measurements for diffusion of AlexaFluor647-labeled streptavidin in DMSO:glycerol solution fitted to the standard formula.

The same procedure has been repeated for the binary mixture of DMSO:glycerol (77%:23%, v/v). Four independent samples with the same ratio of DMSO and glycerol and 0.5 *nM* AlexaFluor647-labeled streptavidin were prepared. For each sample we performed two FCS measurements at two different positions. Figure 12 shows the FCS curves of four measurements (one of the two measurements for each sample). A strong variation of diffusion time and exponent of anomalous diffusion was observed, indicationg the heterogeneneity of the system at larger scales. For curves shown in this figure, the exponents of anomalous diffusion were 0.79, 0.91, 0.87 and 0.52 and the corresponding diffusion times being 26,704 *μs*, 1,072 *μs*, 1,505 *μs* and 6,241 *μs*.

After analysing this heterogeneity at the diffraction-limited scale using FCS, we also performed the spot variation with post-processing to investigate the existence of nano-scale domains in the diffraction-unlimited detection volume.

**Figure 13:**
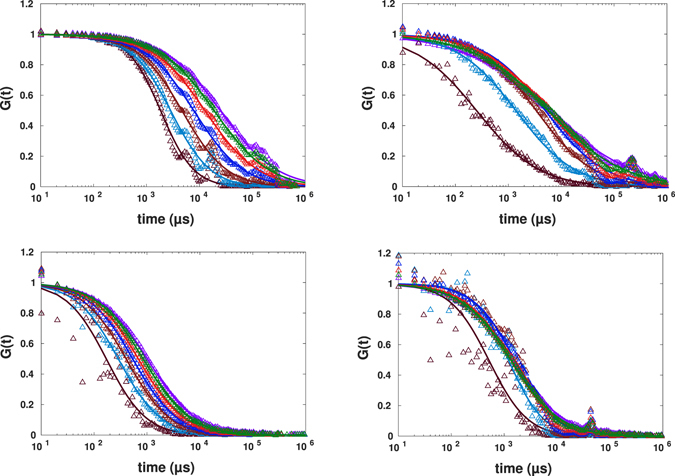
Spot variation FCS with post-processing for four independent measurements in four different samples (*r*_0_ − 0.4*r*_0_ from right to left). The curves were fitted to the standard formula with free fit parameter of exponent of diffusion. The intensity time traces were generated using binning method.

The post-processed data for smaller spot sizes were shown in Fig. 13. Only one of the measurements (shown in the right panel) shows the linear decrease of the diffusion time with the decreasing of the spot size. Three other sets of data show extreme deviations from the behaviour expected for homogeneous systems: Even the exponents of anomalous diffusion obtained for smaller *r*_0_ deviate considerably from the ones obtained from the experimental confocal FCS data. Most of them tend to unity (normal diffusion) for smaller spot sizes, in some cases the fit indicates *α* > 1, which, however, is probably an artefact due to an inappropriate fit function^11^.

**Figure 14:**
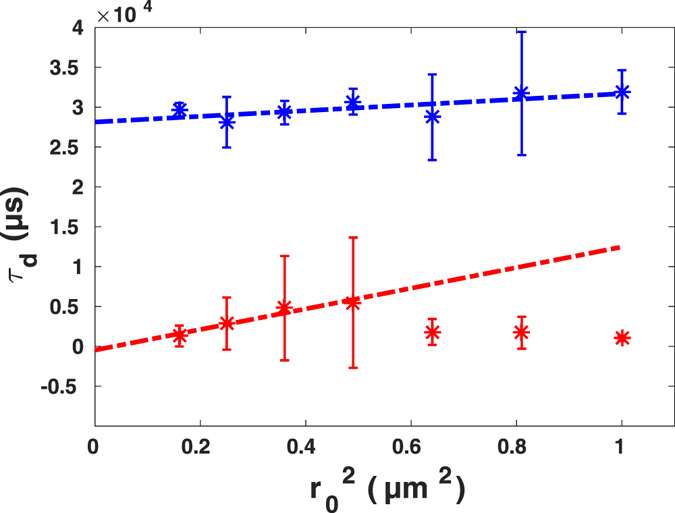
Diffusion time of AlexaFluor647-labeled streptavidin in DMSO:glycerol solution for slow component (blue) and fast component (red) versus spot size, together with corresponding linear fits ${\tau }_{d}\,=\,3,555{r}_{0}^{2}\,+\,28,133$ with *Pearson r* ^2^ = 0.45 and ${\tau }_{d}\,=\,12,941{r}_{0}^{2}\,-\,474$ with *Pearson r* ^2^ = 0.91. Standard deviations were calculated from eight measurements in four sample preparations.

In order to apply the diffusion law analysis, one can not simply average the diffusion times resulting from the different exponents of anomalous diffusion, different exponents for each initial measurement and the spot variated data. Taking into account a binary nature of the system, we performed two-component fits for the all original and spot variated FCS curves. Although the two components were suboptimal, requiring higher number of components, our analysis indicates the existence of the slow component with the diffusion time of 31,907 ± 2,726 *μs*, which is roughly ten times larger than the diffusion time in pure DMSO, which is in agreement with previous results^13^. The spot size dependence of slow (blue) and fast (red) diffusion time is in Fig. 14. The diffusion time for the slow diffusion did not follow the expectation for a homogeneous system, and the intercept of a linear fit was 28,133 ± 2,726 *μs* indicating the existence of nano-domains within the confocal volume, in which tracer molecules spends much longer time compared to the free diffusion in the DMSO phase as indicated by the fast time component of 1,019 ± 3,360 *μs*. This result in principle could be considered as an experimental proof of the ability of the method to detect nano-domains.

The analysis of the fast component for larger spot sizes is very inaccurate due to the overall very slow decay of curves. However, when the size of spot is decreased using spot variation, the influence of slow diffusion due to nano-domains is decreasing in most of the FCS curves. For <0.5*r*_0_ the behavior of the diffusion time is similar to those for pure DMSO. Excluding the data for larger effective spot sizes from the linear fit results in intercept being: −474 ± 3,323 *μs*.

### *In vivo* experiments

**Figure 15:**
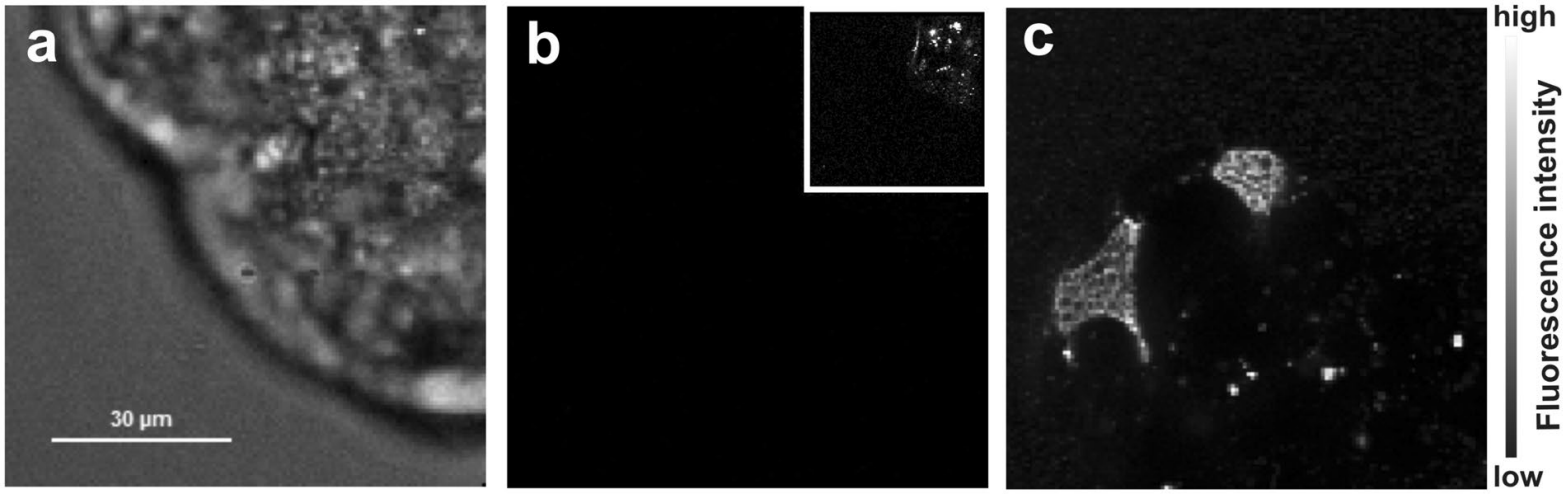
(**a**) Bright field image of chosen cell region to be injected, (**b**) fluorescence image of cell region before injection, (inset) fluorescence image before injection boosted 40 times for visibility of autofluorescence, (**c**) fluorescence image of cell region after injection showing AlexaFluor647 fluorescence in two different injection points.

As an application of our method to living cell studies, we have performed intracellular FCS measurements. The microscopic picture corresponding to successful injection of AlexaFluor647-labeled streptavidin is given in Fig. 15.

**Figure 16:**
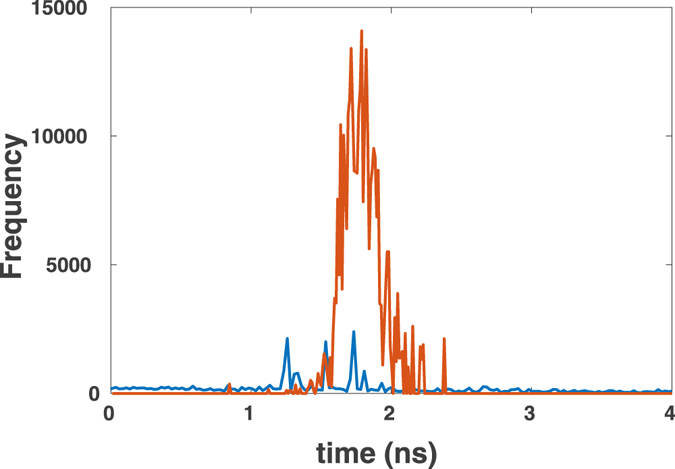
Fluorescence lifetime histograms extracted from both images, before (blue) and after (red) injection. Additional photon counts after injection and their lifetime distribution indicate successful injection process of AlexaFluor647-labeled streptavidin.

To check the successful injection process, fluorescence lifetime histograms before and after the injection process were recorded. Results shown in Fig. 16 indicate the broad lifetime distribution of autofluorescence (blue) before injection. After injection of AlexaFluor647-labeled streptavidin, the lifetime histogram is enhanced and centred around 1.5 *ns*. The biexponential fluorescence decay analysis (not shown) resulted in average decay time 1.6 *ns*. This is in agreement with the reported lifetime for AlexaFluor647-labeled streptavidin in buffer solution indicating a biexponential fluorescence decay with an average lifetime of 1.5 *ns* ^16^.

**Figure 17:**
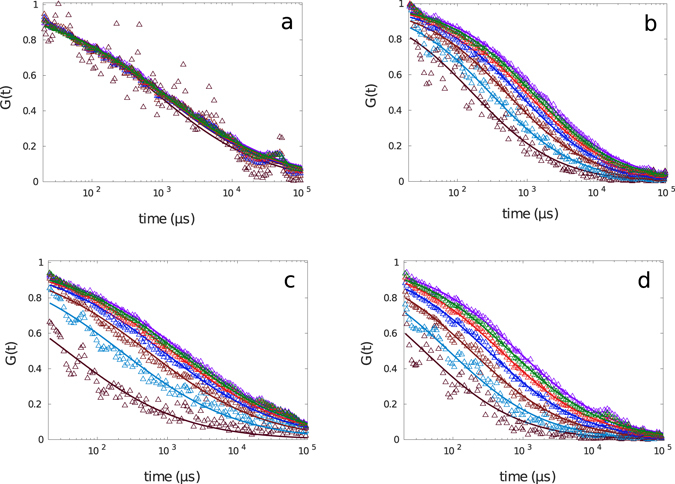
Experimental results for FCS calculated from smoothed FCS curves with *σ* = 3 *μs* (circles) together with results for mimicked spot variation FCS (with spot size varying from *r*_0_ to 0.4*r*_0_ from right to left) for the diffusion of AlexaFluor647-labeled streptavidin in a living cell in different preparations fitted to the standard formula.

After injection, we performed intracellular FCS measurements. The calculated autocorrelation functions are shown in Fig. 17. Similar to the case the binary mixture of DMSO:glycerol, we have observed different results for four different cell preparations. Plotted in Fig. 17 are the FCS curves corresponding to four different measurements together with post-processed spot variation data, respectively. The exponents of anomalous diffusion changed considerably from one to another measurement and were 0.49, 0.68, 0.50 and 0.62 and the corresponding diffusion time being 1,199 *μs*, 1,496 *μs*, 1,770 *μs* and 903 *μs*. The spot variation FCS results apparently differ from one to another sample and except for one measurement, the results unravel presence of either transient interaction with other macromolecules or trapping due to geometrical confinement bellow the diffraction limit.

**Figure 18:**
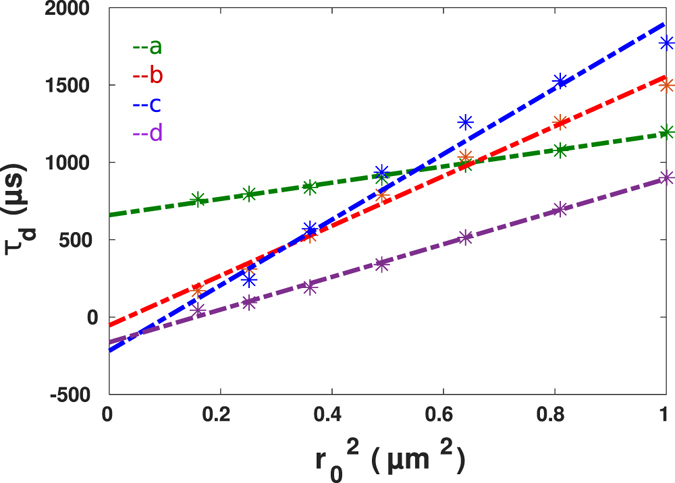
The diffusion law analysis for four living cell FCS measurements (shown in Fig. 17) together with the linear fits.

The dependence of the effective diffusion time on the spot size is shown in Fig. 18. These data and the corresponding linear fits indicate different bevaviour of diffusion time with change of spot size. Positive and negative intercepts observed in diffusion law analysis for different independent experiments underlining the existence of a complex intracellular-environment. Here, streptavidin seems to interact with other macromolecules transiently or its diffusion is hindered due to existing nano-barriers. Similar experimental results were observed recently by using STED-FCS^17^. Generally, in living cells intracellular organelles and membrane systems as well as cytoskeleton elements form a dynamic three-dimensional maze through which tracer molecules have to find their way, leading to hopped diffusion. This kind of trapped diffusion can be the result of transient protein-specific interactions with intracellular components. Such transient complexes would then diffuse slower or could be locally immobilized or geometrically repulsed^18–21^.

## Conclusions

We have proposed a method of mimicking the spot variation fluorescence correlation spectroscopy (SV-FCS) using post-processing of recorded photon counts from ordinary diffraction-limited confocal FCS measurements. Recorded photons counts from FCS measurements are transformed to smoothed intensity traces using kernel smoothing method with the double-sided exponential kernel or to a piecewise-continuous intensity trace using binning. The resulting intensity trace is then non-linearly transformed. The transformed trace corresponds to the one which would be obtained for the same particles’ trajectories for a smaller confocal volume, whose waist is controlled by a free parameter *r*_*c*_ of the transformation. The method is checked in extensive numerical simulations, and is applied to experimental situations.

Applying the method to diffusion of AlexaFluor647-labeled streptavidin in PBS buffer solution and in buffer crowded by 30% PEG1500, we have shown that for these homogeneous systems the effective diffusion coefficient does not depend on the mimicked spot size and equals to the one obtained in normal FCS, which gives an additional (although indirect) support for the validity of our approach. Applying the method to the diffusion of the same tracer in DMSO and the binary mixture of DMSO:glycerol we tested the method’s ability to detect nano-domains at sub-diffraction level.

Finally, the same procedure was applied for the case of diffusion of streptavidin in living cells. As expected, the results of such application indicate strong inhomogeneity of the intracellular medium.

The advantage of the approach is that SV-FCS can be performed with the standard FCS setup and without the need to repeat the measurements preventing the errors due to recalibration, drifts and thermal instabilities. The limitation of the method is that it requires low concentration of tracers, below 1 *nM* for typical size of confocal volume, i.e. may need longer measurement times. We have shown that 100 *s* data acquisition time is sufficient to obtain reliable results for effective spot sizes down to 1/3 of the initial one. Increasing the measurement time even more would lead to less noisy data and allow to extract the effective FCS curves for the spot sizes an order of magnitude smaller than initial spot size. SV-FCS using data post-processing is an experimentally simple and straightforward single-molecule technique feasible for studying the subcelluar organisation of living cells.

## Methods

### Simulation method

In our numerical simulations for normal and anomalous diffusion, the trajectories of each tracer were obtained from their coordinates simulated as three independent one-dimensional Brownian and fractional Brownian motions along each of the coordinate axes. The trajectories for normal diffusion were generated using the Box-Muller method^22^. For the case of anomalous diffusion, the trajectories were calculated as integrating the fractional Gaussian noise generated using Lowen’s method^23^.

### Experiment details

The measurements have been performed on a MicroTime 200 time-resolved confocal microscope (PicoQuant, Berlin, Germany) at the Physical Chemistry group of University of Potsdam. Samples were excited by a laser diode (LDH-P, PicoQuant) with wavelength of 635 nm, repetition rate of 20 MHz and pulse width of 70 ps. The excitation beam was focused by a 100x/NA1.4 oil immersion objective (Olympus, Hamburg, Germany). Fluorescence light emitted from dye passes through the dichroic mirror (z467/635rpc, AHF Analysentechnik, Tübingen, Germany), is guided through a 50 *μ*m pinhole and is recorded by single photon avalanche diode (SPAD)(SPCM-AQR-13, Perkin-Elmer, USA). To calculate the autocorrelation functions and for analysis we used Matlab R2016a (9.0.341369). In order to obtain parameters *r*_0_ and *s* entering the apparatus function, we performed a calibration experiment with Atto655 diffusion in water at 293.15 K, for which the diffusion is normal and the diffusion coefficient is known: *D* = 392 *μ*m^2^/s^24^. Calibration leads to the values of parameters *r*_0_ = 0.248 *μ*m and *s* = 6.5.

### Living cell preparation

In order to perform intracellular FCS measurements, salivary gland tissue of the American cockroach *Periplaneta americana* was dissected in physiological saline. Subsequently, the tissue was attached to a glass cover slip using the tissue adhesive Vectabond (Axxora, Lörrach, Germany) and the recording chamber was then mounted on the microscope stage. For dye injection, micropipettes with tip sizes of approximately 2 *μ*m were prepared from glass tubes with filaments (GB150F-10, Science Products, Hofheim, Germany) using a micropipette puller (P-97, Sutter Instruments, Novato, USA). Micropipettes were loaded with 100 nM streptavidin labeled with AlexaFluor647 and injection into salivary gland cells was performed with an injection system (FemtoJet, PatchMan NP2, Eppendorf, Hamburg, Germany) under microscopic view (IX71, Olympus, Hamburg, Germany).
